# Spectroscopic parameters of the cuticle and ethanol extracts of the fluorescent cave isopod *Mesoniscus graniger* (Isopoda, Oniscidea)

**DOI:** 10.3897/zookeys.515.9395

**Published:** 2015-07-30

**Authors:** Andrei Giurginca, Vladimír Šustr, Karel Tajovský, Maria Giurginca, Iulia Matei

**Affiliations:** 1 ”Emil Racovita” Institute of Speleology, 13 Septembrie Str., no. 13, Sector 5, 050711 Bucharest, Roumania; 2 Institute of Soil Biology, Biology Centre, Czech Academy of Sciences, Na Sádkách 7, 370 05 České Budějovice, Czech Republic; 3 Polytechnica University Bucharest, Roumania; 4 Department of Physical Chemistry, Faculty of Chemistry, University of Bucharest, Roumania

**Keywords:** *Mesoniscus graniger*, autofluorescence, molecular spectroscopy, β-carboline and coumarine derivatives

## Abstract

The body surface of the terrestrial isopod *Mesoniscus graniger* (Frivaldsky, 1863) showed blue autofluorescence under UV light (330–385 nm), using epifluorescence microscopy and also in living individuals under a UV lamp with excitation light of 365 nm. Some morphological cuticular structures expressed a more intense autofluorescence than other body parts. For this reason, only the cuticle was analyzed. The parameters of autofluorescence were investigated using spectroscopic methods (molecular spectroscopy in infrared, ultraviolet-visible, fluorescence, and X-ray fluorescence spectroscopy) in samples of two subspecies of *Mesoniscus graniger* preserved in ethanol. Samples excited by UV light (from 350 to 380 nm) emitted blue light of wavelengths 419, 420, 441, 470 and 505 nm (solid phase) and 420, 435 and 463 (ethanol extract). The results showed that the autofluorescence observed from living individuals may be due to some β-carboline or coumarin derivatives, some crosslinking structures, dityrosine, or due to other compounds showing similar excitation-emission characteristics.

## Introduction

Among arthropods, the fluorescence of body surface was firstly reported in scorpions. The intensity of the fluorescence increased with the hardening of the cuticle (Pavan and Vachon 1954, Lawrence 1954). However, other invertebrates, e.g. cockroaches (Neff et al. 2000) and marine as well as freshwater crustaceans (Zimmer et al. 2002, Mazel 2005, Haug et al. 2011) also showed fluorescence.

Scorpions emit visible light (400–700 nm) under UV radiation (Fasel et al. 1997). In *Euscorpius italicus* (Herbst, 1800) the fluorescent substance is concentrated in the thin hyaline layer of the cuticle and is insoluble in water below 100 °C as well as in other solvents such as ethyl ether, chloroform, acetone, benzene, toluene, and methanol (Pavan and Vachon 1954). However, the fluorescent substance may be partly soluble in alcohol, in which scorpions are preserved (Wankhede 2004).

Stachel et al. (1999) determined the soluble fluorescent compound from scorpion cuticle as an alkaloid β-carboline using separation by thin layer chromatography and compound identification by nuclear magnetic resonance (NMR) and high performance liquid chromatography (HPLC). β- carboline was also reported from the human cataracts (Wankhede 2004). β-carboline derivatives, some with hallucinogenic effects, are known from some plants (Hadley et al. 1974, Cao et al. 2007).

It is assumed that more than one fluorescent compound may be present in scorpions. 7-hydroxy-4-methylcoumarin was detected as another fluorescent compound in an extract of scorpion cuticle by Frost et al. (2001) using HPLC for separation and detecting the fluorescence by fluorimetry. The substance was identified by gas-chromatography mass-spectrometry (GCMS). 7-hydroxy-4-methylcoumarin is often used as fluorogenic marker in enzyme assays (also known as 4-methylumbelliferone - Miller et al. 1998, Gee et al. 1999). Coumarin derivatives were found mainly in plants, but also in prosobranch molluscs and in the scent glands of beavers (Murray et al. 1982). Another possible fluorescent compound found in the cuticle of arthropods is resilin. It is a very elastic protein with an irregular structure: its randomly coiled chains are crosslinked by di- and tri-tyrosine links (Elvin et al. 2005). In the cockroach *Periplaneta americana* (Linnaeus, 1758) fluorescence of the ligaments of the tarsus containing resilin was observed (Neff et al. 2000).

The autofluorescence of the cuticle of the cave isopod *Mesoniscus graniger* (Frivaldsky, 1863) was found during analysis of the content of its digestive tract under fluorescent microscope (Giurginca et al. 2012). *Mesoniscus graniger* is the first terrestrial isopod in which autofluorescence was observed from the entire body. Autofluorescence was recorded in the isopod *Nataldillo burnupi* (Collinge, 1917) by Lawrence (1954) but only a weak one from the sternites; the chemical compound responsible for this isopod autofluorescence is not yet known. The aim of our study was therefore, to describe the autofluorescence in detail using microscopic observations and to measure spectroscopic characteristics of the substances responsible for the *Mesoniscus graniger* cuticle autofluorescence. Only the cuticle was investigated; although in the ethanol extracts, there might be fluorophore products resulting from the dissolution of the soft tissues, in our opinion the cuticle (the exoskeleton) contributed the most of the fluorescent signal. Moreover, the cuticle of *Mesoniscus* has not enough transparency to allow the observation of the soft tissues fluorescence.

## Material and methods

### Material

Living as well as individuals of *Mesoniscus graniger* preserved in ethanol, were used in our study. Living animals were sampled for epifluorescent microscopy in the Slovak Karst National Park (Domica and Ardovská caves). The individuals stored in ethanol used for spectroscopic analyses were collected in the Romanian Karst: the Cernişoara Valley, 20 individuals corresponding to the subspecies *Mesoniscus graniger graniger* (Frivaldsky, 1863) (labelled in the following analyses as G) and from the Sighiştelului Valley, 16 individuals corresponding to the subspecies *Mesoniscus graniger dragani* Giurginca, 2003 (labelled in the following analyses D). In order to assess if the autofluorescence is present in the entire range of *Mesoniscus graniger*, we used individuals from the Petnička Pećina (Valjevo, Serbia) and for assessing the presence of this feature in both species of the genus *Mesoniscus*, we tested the individuals of *Mesoniscus alpicola* (Heller, 1858) from the Falkensteinhöhle (Niederösterreich, Austria).

### Fluorescence imaging

Photographs of living fluorescent individuals of *Mesoniscus graniger* were recorded with the Olympus XZ61 stereomicroscope equipped with the Olympus DP20 camera and the Hoya UV (0) photographic filter using the Helling UV-Inspector 385 lamp (365 nm) as a source of excitation light. Animals were placed in a refrigerator for a minute to reduce their movement before taking pictures. Images obtained in different focal planes were stacked by the Helicon Focus 5.3 software (Helicon Soft, Ltd.) to obtain a large depth of focus for the resulting photos. Details of fluorescent body surface of *Mesoniscus graniger* were documented on the Olympus BX 60 fluorescent microscope equipped with the Olympus DP50 camera. The Olympus U-MWU mirror unit (330-385 nm exciter filter and BA420 barrier filter) was used.

Under field conditions, the autofluorescence of living animals was documented with the Canon EOS 600D camera under the excitation light of the Helling UV-Inspector 385 lamp in the Ardovská Cave (Slovakia).

The autofluorescence of *Mesoniscus graniger* from Serbia and that of *Mesoniscus alpicola* was confirmed under the Bactericide Lamp LBA 55W (253.7 nm) and the First Light Illuminator-System Biodoc (302 nm). No spectral analyses were performed on the samples of *Mesoniscus graniger* from Serbia and on the samples of *Mesoniscus alpicola*.

### Spectroscopic analyses

**Sample preparation**:

The samples preserved in 75% ethanol were filtered in order to separate the solid from the liquid phase (ethanol extract). The solid phase was air dried and stored in Petri-type laboratory vessels; the liquid phase was kept in Erlenmayer-type laboratory vessels.

**Apparatus and investigation methods**:

For the analyses of samples (G solid phase, G ethanol extract, D solid phase, and D ethanol extract) we used molecular spectroscopy techniques in the infrared (IR) (middle – MID and near – NIR), ultraviolet-visible (UV-VIS) and fluorescence (FP) range. In addition, a part of each sample was analyzed by X-ray fluorescence spectroscopy (XRF).

For the **IR analysis**, we used the Bruker Optics Tensor 27 spectrometer, with Opus 4.2 specialized software, in the 500–4000 cm^-1^ range. The analysis used the spectral KBr technique with a device for micro-pellets. The IR analysis was used for the solid samples and the ethanol extracts.

For the **UV-VIS and NIR analysis**, we used the UV-VIS-NIR-620 apparatus (Jasco, Japan) with 10 ml quartz cells for the liquid phase and with the ILN-725 diffuse reflection accessory for the solid phase, in the 200–2500 nm range. The apparatus has a monochromator and photoelectric cells corresponding to the investigated domains (UV = 200–400 nm, VIS = 400–800 nm and NIR = 800–2000 nm). Although the NIR region is a part of the IR spectroscopy, for constructive reasons it was included in this apparatus, the energy source being more powerful than that used for the IR range. The UV-VIS and NIR analysis was used for the solid samples and the ethanol extracts.

For the **molecular fluorescence analysis**, we used the FP6500 and FP6300 spectrofluorimeters (Jasco, Japan) using 10 ml quartz cells for the liquid phase and special tanks with quartz window for the solid phase, in the 200–800 nm range. Specific wavelengths were used for excitation in the UV-VIS range, with sources specific to each spectral region (UV and VIS) and the emission spectra were registered. The FP analysis was used for the solid samples and the ethanol extracts.

For the **XRF (X-ray fluorescence) analysis**, a part of each sample was grounded in an agate mortar and, subsequently, loaded into small plastic cylinders and XRF-analyzed on a Horiba XGT-7000 X-ray Analytical Microscope. The XRF analysis was used only for the solid samples.

## Results and discussion

### Autofluorescence microscopy

**Figure 1.**
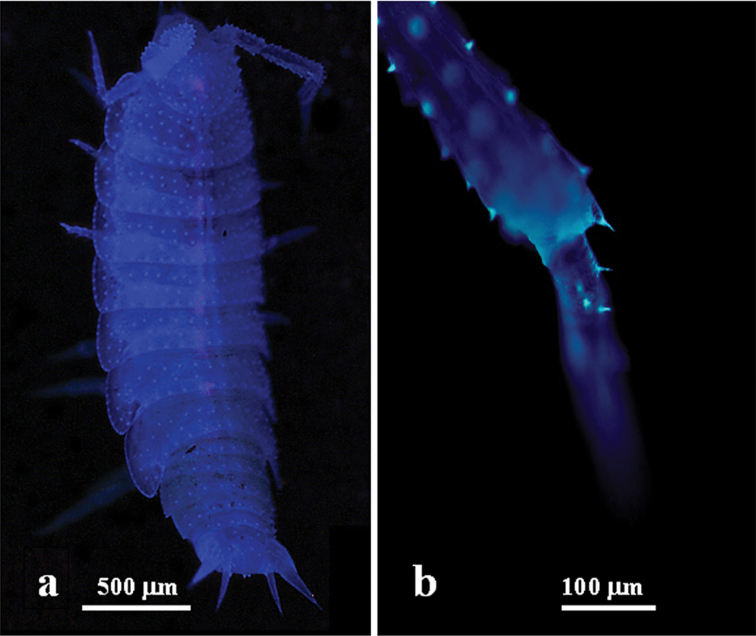
Autofluorescence of the body of *Mesoniscus graniger* under UV light. **a** stereomicroscope with UV-inspector 385 (excitation light 365nm) **b** detail of the antennae - fluorescence microscope U-MWU mirror unit (330–385 nm).

*Mesoniscus graniger* body surface shows a blue auto-fluorescence when excited with UV light at a wavelength of 365 nm (Fig. 1a) or 330–385 nm (Fig. 1b). Tubercles on the cuticle surface have a pale blue auto-fluorescence more intense than all the rest of the body surface (Fig. 1a, b).

Autofluorescence was present in all tested specimens of *Mesoniscus graniger* collected from different localities inhabited by this species from the Slovak to the Serbian karst regions. Both subspecies of *Mesoniscus graniger* from Romania (*Mesoniscus graniger graniger* and *Mesoniscus graniger dragani*) show the same intensity of autofluorescence, which is also found in *Mesoniscus alpicola*, the second species of the genus.

### Autofluorescence in the field

Following observations made under laboratory conditions, we tried to document the autofluorescence under field conditions (See Suppl. material 1). As the movie clearly shows, under visible light *Mesoniscus* presents a white color, but under UV light, the body surface shows a blue auto-fluorescence.

### Molecular spectral analysis

**Figure 2.**
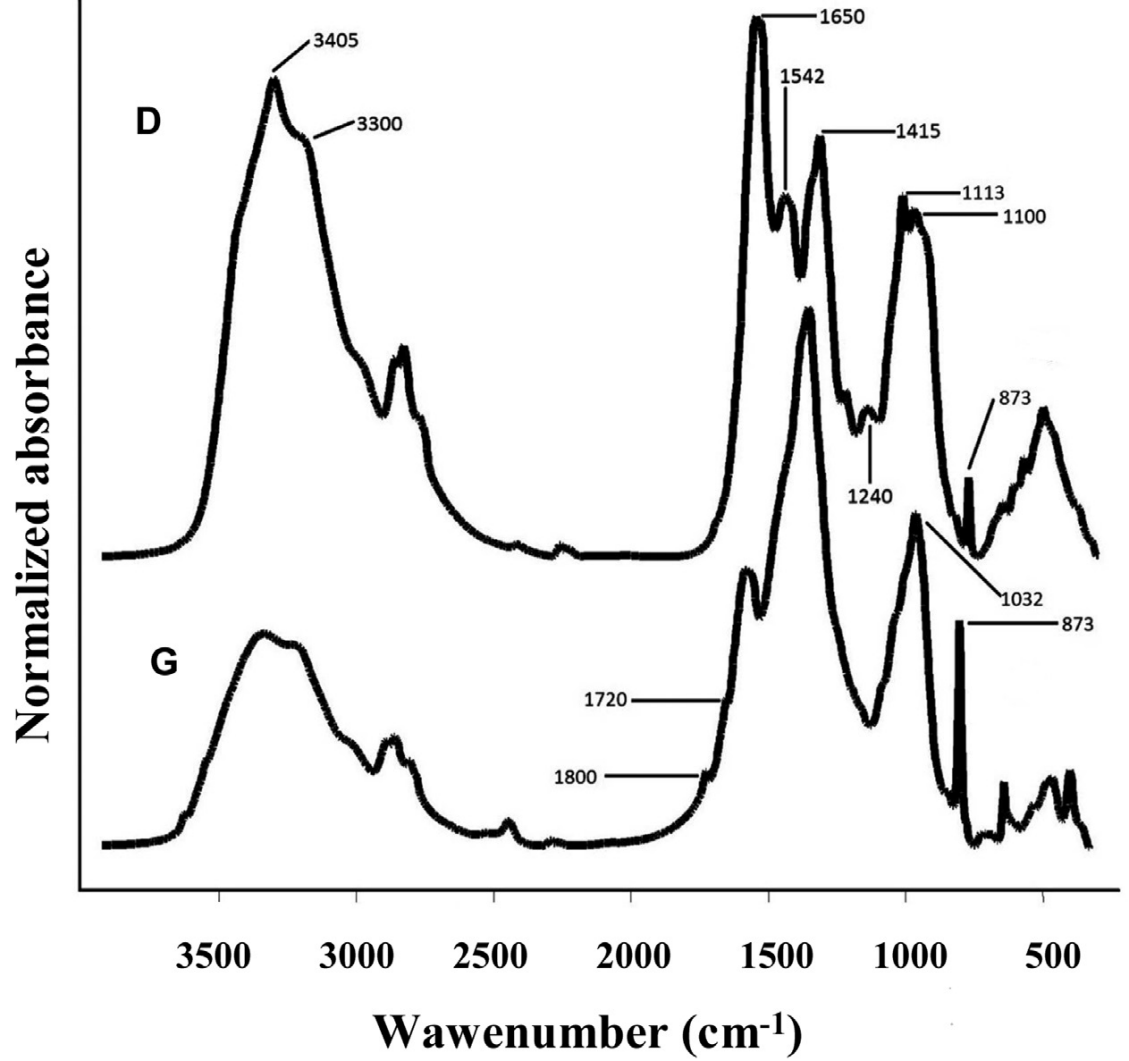
IR spectra of the samples of solid phase of *Mesoniscus graniger graniger* (**G**) and *Mesoniscus graniger dragani* (**D**).

The FT-IR (Fourier Transform Infrared) analysis of the solid phase of both subspecies (Fig. 2D, G) showed a polypeptide structure with characteristic bands at 1650 cm^-1^ (νC=O – amide I), 1542 cm^-1^ (δNH – amide II) and amide III (νC-N-C – 1240 cm^-1^) besides aliphatic (νCH, νCH2) at 2955–2850 cm^-1^ and hydroxyl + amino groups (νOH + νNH) at 3405–3300 cm^-1^, originating in the constitutive amino acids and the glucosamine (Fig. 2D, G) (Balaban et al. 1983). Other FT-IR spectra bands originate from CaCO_3_ (1415 and 873 cm^-1^). The 1113 and 1100 bands are resulting from C-OH and C-NH groups from the N-acetyl glucosamine (chitine) (Fig. 2D).

The FT-IR spectra of the sample of the subspecies *Mesoniscus graniger graniger* (Fig. 2G) presented a series of peculiarities, in which the ageing of the sample must be taken into consideration. A higher content of CaCO_3_ and changes in the peptide structure were recorded, explaining the differences in the spectra: a diminution of the 1650 and 1542 cm^-1^ bands (δNH – amide II) and the disappearance of the 1240 cm^-1^ band (amide III) pointing to the alteration of the polypeptide structure with the involvement of the NH group. Carbonyl/carboxyl structures at 1720 and 1800 cm^-1^ (ketones and/or organic acids), point to a hydrolytic type of oxidative process with the involvement of the NH group from N-acetyl-glucosamine highlighted by the 1032 cm^-1^ band, attributed to the N-CO-C group (Neniţescu 1965). There were no other differences between the bands recorded for the subspecies of *Mesoniscus graniger*.

**Figure 3.**
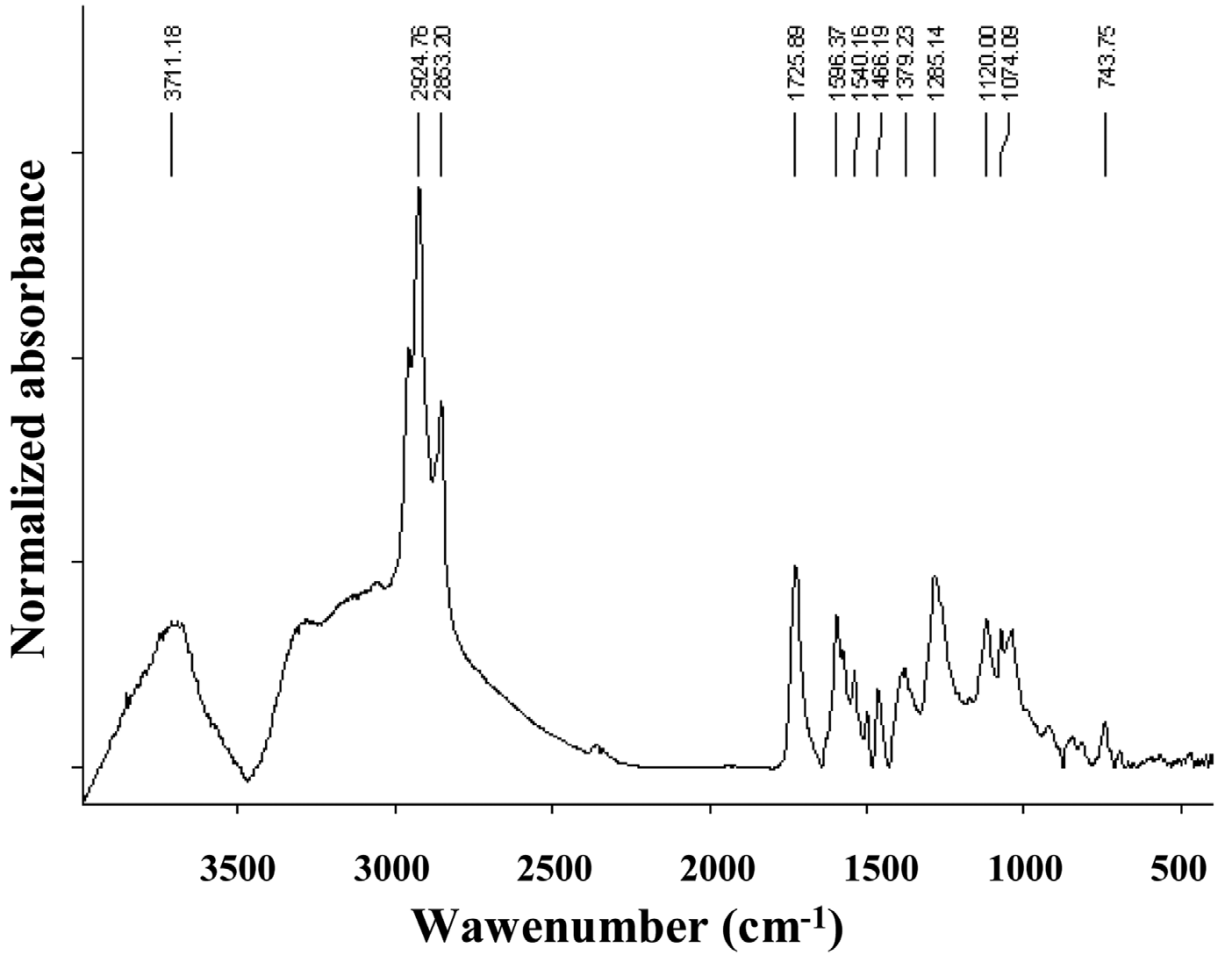
IR spectra of the ethanol extract of *Mesoniscus graniger graniger*.

**The FT-IR analysis of the ethanol extract** of the subspecies *Mesoniscus graniger graniger* showed bands belonging to aromatic fragments and some oxidation compounds (carbonyl group νC = O at 1725 cm^-1^), pointing to a break in the amidic chain proved by the absence of the 1240 cm^-1^ band (amide III). The absence of the 1240 cm^-1^ band might be due to the insolubility of some compounds (Fig. 3).

The presence of Ca, already inferred by the IR analysis, was confirmed by the **XRF analysis**, the Ca content (weight %) being 43.83% in sample G and 16.25% in sample D (expressed as Ca^2+^).

**Figure 4.**
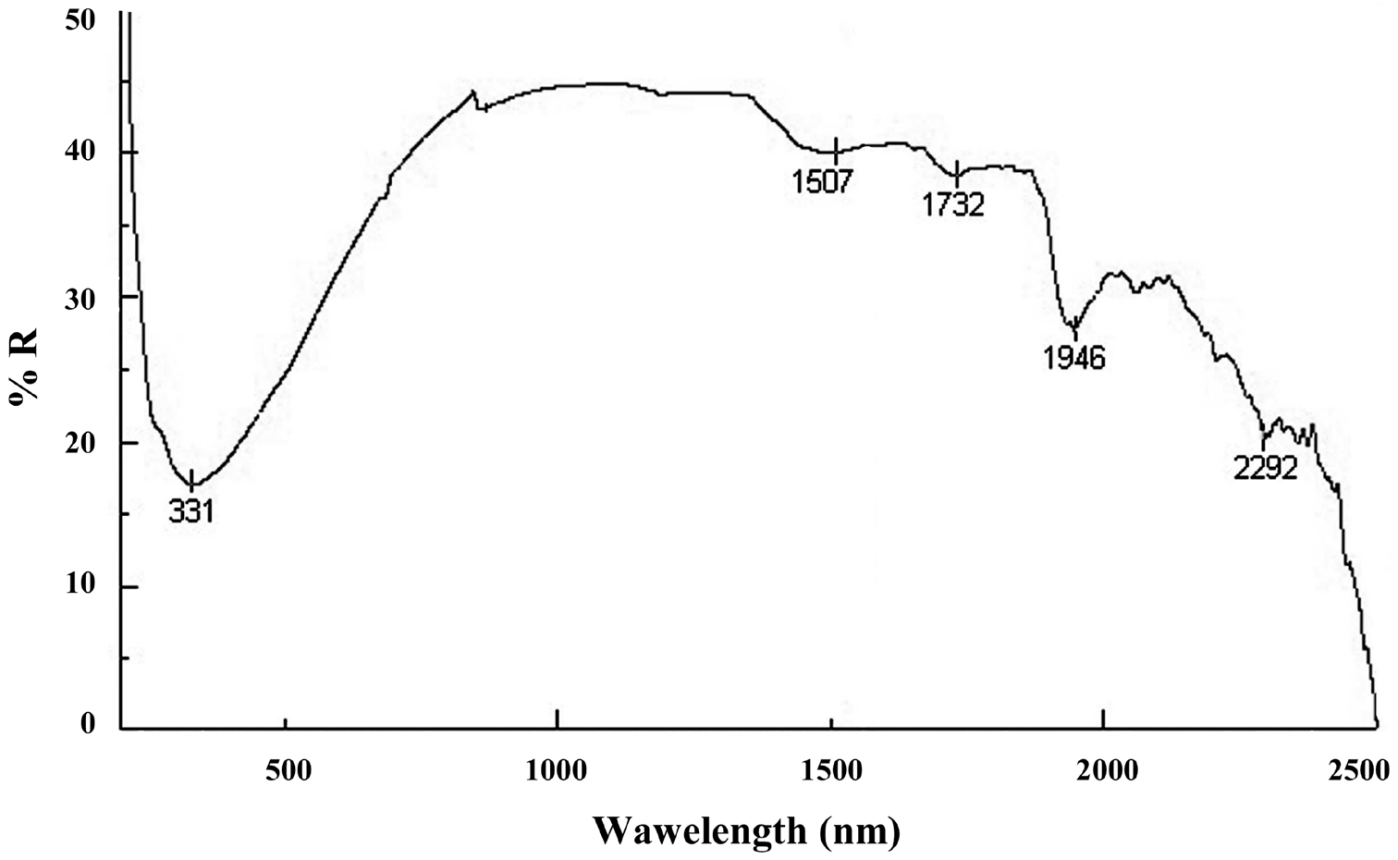
UV-VIS-NIR spectra of the sample of solid phase of *Mesoniscus graniger dragani* (% R = percent reflectance).

**The analysis in the UV-VIS-NIR domains** of the samples solid phase undertaken on the material of the subspecies *Mesoniscus graniger dragani* only (D samples) showed several characteristic bands (Fig. 4): a wide band situated between 200 and 600 nm, with a maximum at 331 nm, pointing to a combination between the transitions π→π^*^ + n→π^*^ and an extended conjugation system (Balaban et al. 1983); the 1507 nm band emphasizing the presence of intra/intermolecular hydrogen bonds formed with the involvement of the OH and NH groups from peptides and chitin; the bands from 1732, 1946 and 2292 nm come from hydroxylic groups (νOH + δOH) present in the polypeptide chain, but also in chitin (Egawa et al. 2003, Badea et al. 2008).

**Figure 5.**
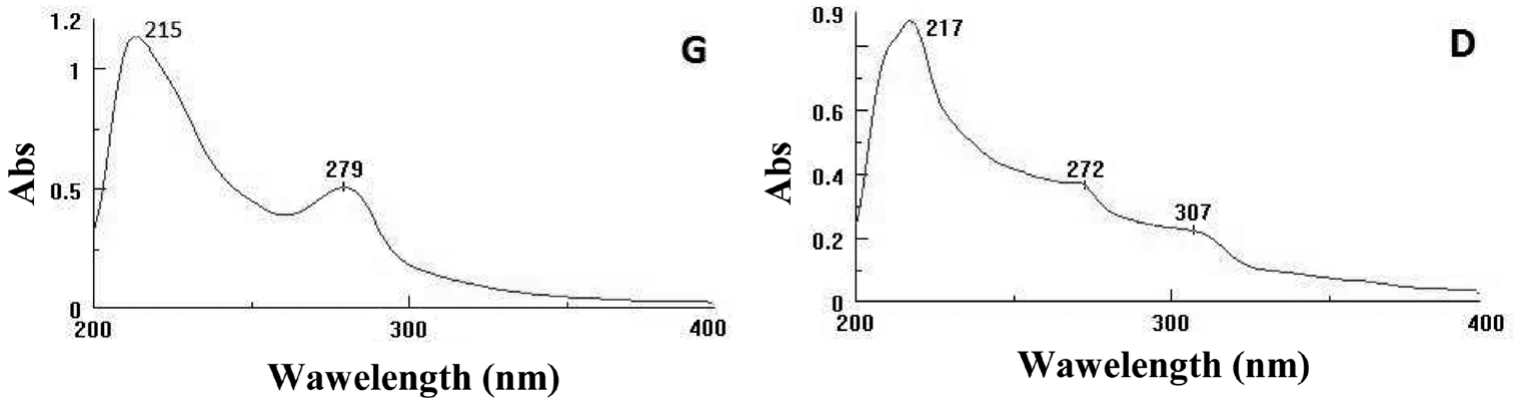
UV-VIS-NIR spectra of the ethanol extracts of *Mesoniscus graniger graniger* (**G**) and *Mesoniscus graniger dragani* (**D**) (Abs = Absorbance units).

The UV-VIS-NIR analysis of the ethanol extracts (Fig. 5D, G) presented only bands characteristic to the π→π^*^ + n→π^*^ transitions in the 210-220 nm belonging to the aromatic structures and n→π^*^ at 275-280 nm (the CONH group from amino acids). The D sample showed a weak band coming from conjugated structures which led to the yellow colour of the solution.

**Figure 6.**
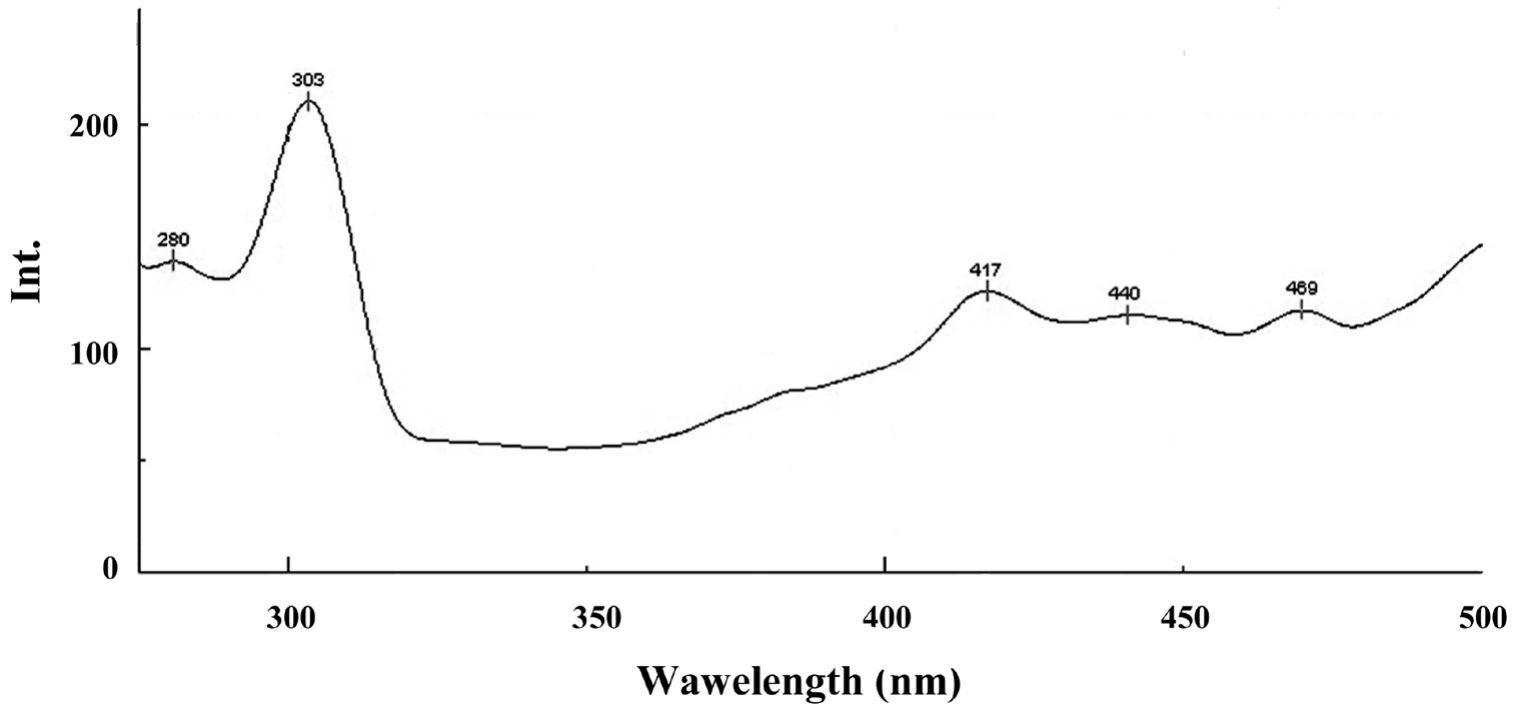
FP spectra of the sample of solid phase of *Mesoniscus graniger dragani* (λex = 265 nm) (Int.- intensity of the peak).

**The molecular fluorescence analysis (FP)** of the samples of solid phase of *Mesoniscus graniger dragani* obtained by excitation at 265 nm (Fig. 6) showed several characteristic bands: the 280 nm band can be attributed to the phenylalanine (Lakowicz 2002); the 303 nm band was expressed due to the presence of tyrosine (Lakowicz 2002); the 417 and 440 nm bands are attributed to some crosslinking structures (possibly lipids from membranes) and to the dityrosine (Dolgin et al. 2009, Ross et al. 2002); the 469 band points to the presence of a β-carboline derivative, taking into account the light blue - blue colour of the fluorescent emission (Stachel et al. 1999).

**Figure 7.**
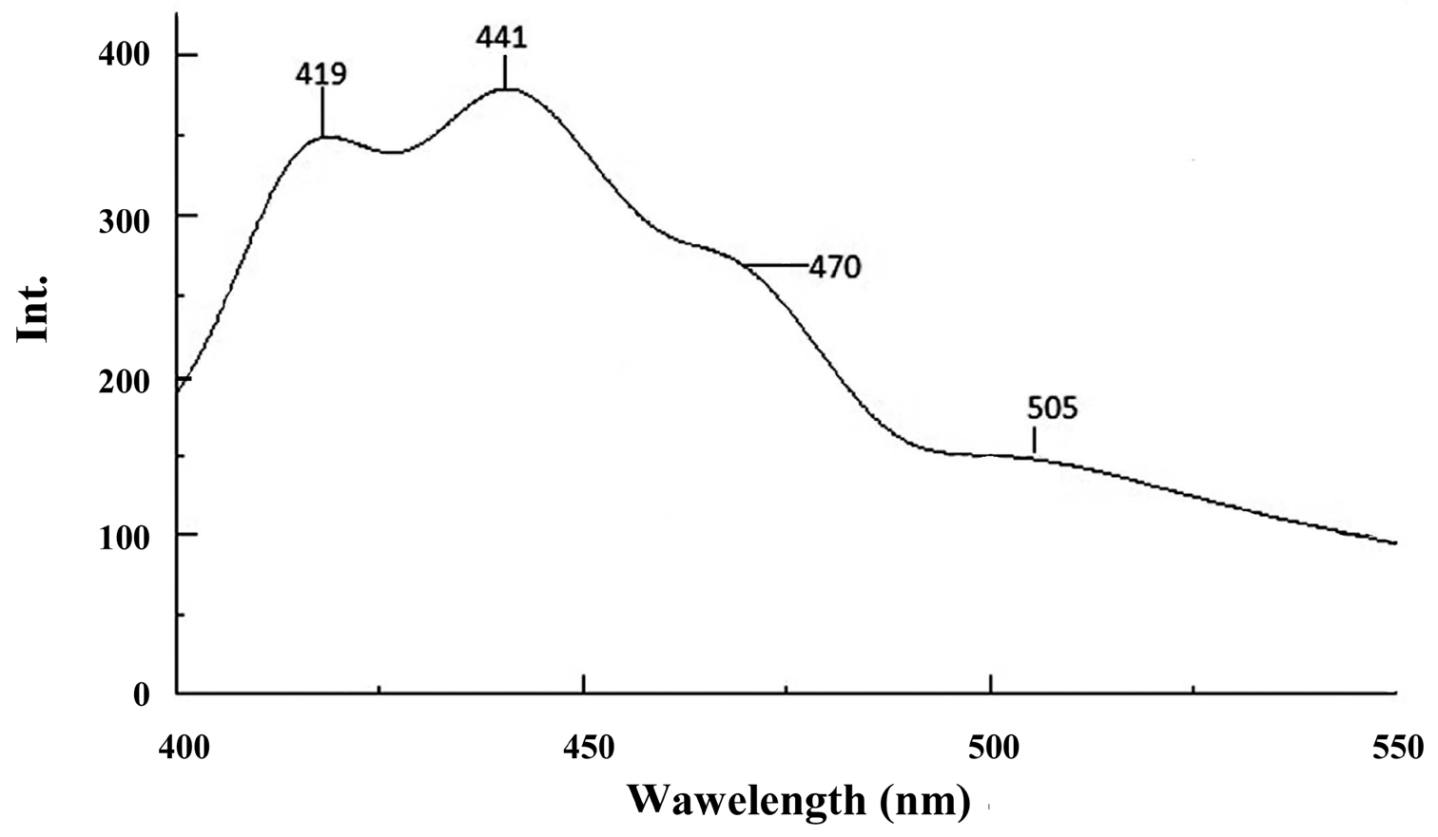
FP spectra of the sample of solid phase of *Mesoniscus graniger dragani* (λex = 380 nm) (Int.- intensity of the peak).

The FP analysis with excitation at 380 nm (Fig. 7) led to the emission spectra with bands at 419 nm and 441 nm responsible to the crosslinking structures (containing bonds between molecular chains with the involvement of aromatic structures) and dityrosine, 470 nm to β-carboline derivative and 505 nm corresponding to the fluorophore structure with extended conjugation resulting more probably from lipid oxidation (Sokolov et al. 2002).

The molecular fluorescence analysis of the ethanol extracts were obtained by excitation at 280, 350 and 380 nm, the colour of the emission being light blue - blue (see Table 1).

**Table 1.** The emission bands of the ethanol extracts.

	D	G
λ excitation (nm)	λ emission (nm)	λ emission (nm)
280	317	313
350	420	435
380	435	463

**Figure 8.**
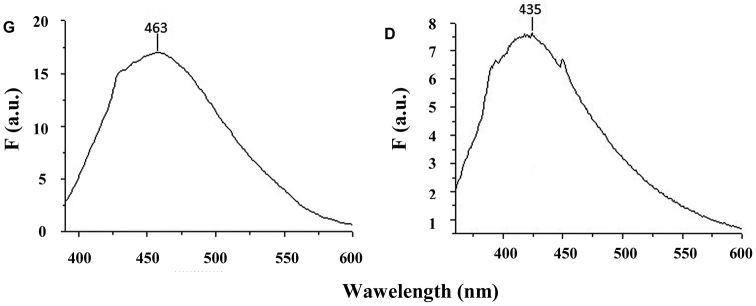
FP spectra of the samples of ethanol extracts of *Mesoniscus graniger graniger* (**G**) and *Mesoniscus graniger dragani* (**D**) samples (λex = 380 nm) (F (a.u.) = fluorescence arbitrary units).

The bands from 313 and 317 nm are attributed to the presence of tyrosine and some aromatic structures with hydroxyl groups (λex = 280 nm) (Ross et al. 2002). The bands from 420 and 435 nm result from crosslinking structures and/or the formation of dityrosine by intra/intermolecular hydrogen bonds (λex = 350 nm) (Valeur 2001, Drezek et al. 2001). The bands from 435 and 463 nm (λex = 380 nm) might be produced by substituted β-carboline compounds (Stachel et al. 1999). The higher intensity of the 463 nm band from G sample point to a higher content of β-carboline derivative, which might be attributed to its formation and accumulation over time in the sample (Fig. 8).

The differences between the emission bands (excitation at 380 nm) of the solid samples and ethanol extracts might be due to the formation of hydrogen bonds with the involvement of the OH groups of the ethanol, emphasizing the influence of the reaction environment, but also its interactions with the chitin and the traces of conjugated lipids. Also, the different solubility in alcohol of the various compounds leads to differences between the emission bands. The molecular fluorescence (FP) tests confirm the data provided by the autofluorescence microscopy, allowing the identification of the β-carboline (beside other aromatic compounds) as the main source of the fluorescence.

The investigations by fluorescence microscopy and by spectroscopic molecular analysis showed the presence of fluorescence in the 330–385 nm excitation domains due to aromatic structures, most probably belonging to the β-carboline type, and changes in the polyamide structure at ageing, changes recorded in *Mesoniscus graniger graniger* and *Mesoniscus graniger dragani*.

The autofluorescence is characteristic for all observed individuals without respect to their geographic origins. It was confirmed in all tested specimens from the entire area inhabited by *Mesoniscus graniger*, both subspecies showing the same intensity, and it was found also in *Mesoniscus alpicola* from elsewhere. Furthermore it was recorded in animals observed in caves as well as in individuals kept in laboratory. The individuals stored for long periods in ethanol in collections retain this property. Accidental contamination of *Mesoniscus graniger* by any fluorescent compounds from the food or by fluorescent microorganisms restricted to certain caves, is challenged by the universal presence of the autofluorescence in all tested populations collected from different caves in various geographic areas.

**Figure 9.**
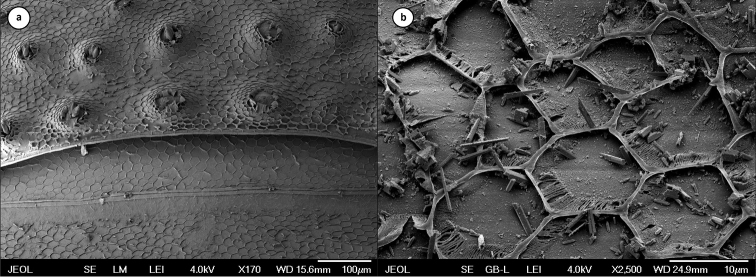
First and second pereionites of *Mesoniscus graniger graniger* showing the position of tubercles (**a**); detail of the honeycomb-like net of scales at *Mesoniscus graniger dragani* (**b**) (after Giurginca et al. 2012 modified).

The very intensively fluorescent structures on the body surface of *Mesoniscus graniger* seem to roughly correspond to some of the structures we observed previously on the body surface of this species using scanning electron microscopy (Giurginca et al. 2012). The cephalon, pereion, and pleon of this species are covered by a series of tubercles connected by finer surface structures similar to scales (Fig. 9). These scales resemble a honeycomb-like net (polygonal structure) and cover almost the entire body surface. Tubercles have a more intense autofluorescence than the net of polygonal scales (Giurginca et al. 2012).

Both the solid and ethanol extract samples contain proteins (polypeptides), chitin (N-acetyl glucosamine) and calcite also identified spectrally in FT-IR and by XRF. It corresponds with general information about the body composition of terrestrial isopods (Wood and Russell 1987, Becker et al. 2005, Giurginca et al. 2010). This composition shows structural changes even when the material is stored in ethanol due to oxidative and enzymatic ageing processes. These aspects of ageing are known for other polypeptide types, for instance for collagen from the human and animal skin, many data coming from studies on new and historical parchments and from leathers tanned with various agents (Badea et al. 2008, Dolgin et al. 2009, Miu et al. 2007). All these changes are described by detailed studies made by IR (MID and NIR), UV-VIS, and FP molecular spectroscopy as well as by other physical and chemical techniques (Badea et al. 2008).

Our observations underline mainly changes of the polypeptides structure by chain alteration (the disappearance of amide III in the case of G sample), crosslinking and the forming of dityrosine and other polycondensated compounds, among which β-carboline due to oxidative processess. We have to stress that β-carboline is present in the body of living animals as a result of their natural ageing and it is not only the result of ageing of material stored in alcohol.

The microscopically observed blue fluorescence of *Mesoniscus graniger* as a response to excitation UV light (about 350 nm) corresponds to the wavelengths range of the blue colour (approximately 450–495 nm after Bruno and Svoronos 2005). Spectroscopic parameters of samples preserved in ethanol, indicated that the autofluorescence emitting blue light observed from the living individuals of *Mesoniscus graniger* may be due to some β-carboline or coumarine derivatives, by some crosslinking structures, dityrosine or due to other compounds showing similar excitation – emission characteristics. The β-carboline or coumarine derivatives were reported to be together responsible for the autofluorescence of scorpions (Stachel et al. 1999, Frost et al. 2001). However, the definitive solution of the problem of the chemical fundament of the autofluorescence of *Mesoniscus graniger* may bring the isolation and analysis of fluorescent compounds as was performed in scorpions by Frost et al. (2001). In a subsequent study, we will follow a non-spectroscopic analytical approach, such as chromatography and other methods.

The functional advantage of invertebrate fluorescence is not yet known regardless of many hypotheses discussed in literature (see Wankhede 2004 or Gaffin et al. 2012). Some observations (Stachel et al. 1999, Wankhede 2004) suggest that the intensity of scorpions autofluorescence is linked to the sclerotisation of cuticle. It is accepted that chemical linking of cuticular proteins can lead to broad-spectrum fluorescence (Wankhede 2004). The dimerization of the cyclic amino acids, tyrosine and tryptophan, leads to the fluorescent compounds resilin and β-carbolines (Stachel et al. 1999). It is possible that fluorescence is not an adaptive feature but just a side effect of a metabolic product with other functional significance or no functional significance at all, as in waste material.

## Supplementary materials

Supplementary material 1 Autofluorescence of living *Mesoniscus graniger* Data type: MPEG video file Explanation note: Living *Mesoniscus graniger* individuals recorded by Canon EOS camera on the cave sediment inside Ardovská Cave (Slovak Karst, Slovakia) under white LED lamp and UV lamp consecutively. This dataset is made available under the Open Database License (http://opendatacommons.org/licenses/odbl/1.0/). The Open Database License (ODbL) is a license agreement intended to allow users to freely share, modify, and use this Dataset while maintaining this same freedom for others, provided that the original source and author(s) are credited.Andrei Giurginca, Vladimír Šustr, Karel Tajovský, Maria Giurginca, Iulia Matei
